# Supplementary material for: Intranasal ChAdOx1 nCoV-19/AZD1222 vaccination reduces shedding of SARS-CoV-2 D614G in rhesus macaques
Source: bioRxiv. 2021 Jan 11:2021.01.09.426058. Preprint. [Version 1] doi: 10.1101/2021.01.09.426058 (PMC7808328; doi:10.1101/2021.01.09.426058)
Supplement: 1 [file NIHPP2021.01.09.426058-supplement-1.pdf]

bioRxiv preprint doi: https://doi.org/10.1101/2021.01.09.426058; this version posted January 11, 2021. The copyright holder for this preprint (which was not certified by peer review) is the author/funder. This article is a US Government work. It is not subject to copyright under 17 USC 105 and is also made available for use under a CC0 license.

Supplementary Materials

Materials and Methods

Ethics statement

The Institutional Animal Care and Use Committee (IACUC) at Rocky Mountain Laboratories provided all animal study approvals, which were conducted in an Association for Assessment

bioRxiv preprint doi: https://doi.org/10.1101/2021.01.09.426058; this version posted January 11, 2021. The copyright holder for this preprint (which was not certified by peer review) is the author/funder. This article is a US Government work. It is not subject to copyright under 17 USC 105 and is also made available for use under a CC0 license.

and Accreditation of Laboratory Animal Care (AAALAC)-accredited facility, following the basic principles and guidelines in the Guide for the Care and Use of Laboratory Animals 8th edition, the Animal Welfare Act, United States Department of Agriculture and the United States Public Health Service Policy on Humane Care and Use of Laboratory Animals.

Animals were kept in climate-controlled rooms with a fixed light/dark cycle (12-hours/12-hours). Hamsters were co-housed in rodent cages, fed a commercial rodent chow with ad libitum water and monitored at least once daily. Rhesus macaques were housed in individual primate cages allowing social interactions, fed a commercial monkey chow, treats and fruit with ad libitum water and were monitored at least twice daily. Environmental enrichment for rhesus macaques consisted of a variety of human interaction, commercial toys, videos, and music. The Institutional Biosafety Committee (IBC) approved work with infectious SARS-CoV-2 virus strains under BSL3+ conditions. All sample inactivation was performed according to IBC approved standard operating procedures for removal of specimens from high containment.

Generation of ChAdOx1 nCoV-19 vaccine

ChAdOx1 nCoV-19 was designed as previously described[8]. Briefly, the S protein of SARS-CoV-2 (GenBank accession number YP_009724390.1) was codon optimized for expression in human cell lines and synthesized with the tissue plasminogen activator (tPA) leader sequence at the 5' end by GeneArt Gene Synthesis (Thermo Fisher Scientific). The sequence, encoding SARS-CoV-2 amino acids 2-1273 and tPA leader, was cloned into a shuttle plasmid using InFusion cloning (Clontech). The shuttle plasmid encodes a modified human cytomegalovirus major immediate early promoter (IE CMV) with tetracycline operator (TetO) sites, poly adenylation signal from bovine growth hormone (BGH). ChAdOx1 nCoV-19 was prepared using Gateway® recombination technology (Thermo Fisher Scientific) between this shuttle plasmid and the

bioRxiv preprint doi: https://doi.org/10.1101/2021.01.09.426058; this version posted January 11, 2021. The copyright holder for this preprint (which was not certified by peer review) is the author/funder. This article is a US Government work. It is not subject to copyright under 17 USC 105 and is also made available for use under a CC0 license.

ChAdOx1 destination DNA BAC vector[38] resulting in the insertion of the SARS-CoV-2 expression cassette at the E1 locus. The ChAdOx1 adenovirus genome was excised from the BAC using unique PmeI sites flanking the adenovirus genome sequence. The virus was rescued and propagated in T-Rex 293 HEK cells (Invitrogen). Purification was by CsCl gradient ultracentrifugation. Virus titers were determined by hexon immunostaining assay and viral particles calculated based on spectrophotometry[39,40]

Study design animal experiments

Syrian hamsters - Syrian hamsters (4–6 weeks old, Envigo Indianapolis, IN) were vaccinated with 100 µl of 2.5 x 10^8 infectious units of vaccine intramuscularly or 50 µl of 2.5 x 10^8 infectious units of vaccine intranasally. Animals were vaccinated 28 days before challenge or exposure. One day prior to virus challenge or exposure animals were bled via the retro-orbital plexus. the direct challenge experiment, 10 animals per group were challenged with 40 µl of 10^4 $TCID_{50}$ SARS-CoV-2/human/USA/RML-7/2020 (MW127503.1) diluted in sterile Dulbecco's Modified Eagle's media (DMEM). In the transmission experiment, 14 unvaccinated donor animals per group were challenged with 40 µl of 10^4 $TCID_{50}$ SARS-CoV-2/human/USA/RML-7/2020 diluted in sterile DMEM. One day later, 14 vaccinated animals per group were co-housed with donor animals at a 2:2 or 1:1 ratio, separated by sex. Four hours later, donor animals were removed from the cage and euthanized. In each experiment, 50% of animals were male and 50% of animals were female. At 5 DPI, four animals were euthanized, and the remaining animals were followed for 21 days post challenge. Weight was recorded daily, and oropharyngeal swabs were taken daily up to 7 days post inoculation in 1 mL of DMEM supplemented with 2% fetal bovine serum, 1 mM L-glutamine, 50 U/ml penicillin and 50 µg/ml streptomycin (DMEM2).

bioRxiv preprint doi: https://doi.org/10.1101/2021.01.09.426058; this version posted January 11, 2021. The copyright holder for this preprint (which was not certified by peer review) is the author/funder. This article is a US Government work. It is not subject to copyright under 17 USC 105 and is also made available for use under a CC0 license.

Upon euthanasia, blood and lung tissue were collected and subsequently analysed for virology and histology.

NHPs – Experimental design was based on a previously reported study[8]. Eight Indian origin rhesus macaques (5F, 3M) between 4-11 years old were sorted by sex, then by weight, and then randomly divided into two groups of four animals. Animal group size was based on initial model development[17]. The vaccine group was vaccinated with 1 ml of ChAdOx1 nCoV-19 using a MAD Nasal™ IN Mucosal Atomization Device (Teleflex, US) at -56 and -28 DPI. Within the control group, 2 animals were vaccinated via the same route with ChAdOx1 GFP, and two animals were vaccinated with ChAdOx1 GFP in 2 ml using an Omron Mesh nebulizer NE-U100. All vaccinations were done with 2.5 x 10^{10} virus particles/animal diluted in sterile PBS. Animals were challenged with SARS-CoV-2/human/USA/RML-7/2020 (MW127503.1) diluted in sterile DMEM on 0 DPI; with administration of 4 mL intratracheally and 1 mL intranasally of 2 x 10^{5} $TCID_{50}$/mL virus suspension. Animals were scored daily by the same person who was blinded to study group allocations using a standardized scoring sheet as previously described[16]. Scoring was based on the evaluation of the following criteria: general appearance and activity, appearance of skin and coat, discharge, respiration, feces and urine output, and appetite. Clinical exams were performed on -56, -49, -42, -28, -21, -14, -7, 0, 1, 3, and 5 and 7 DPI. Nasosorption samples and blood were collected at all exam dates. Nasosorption samples were collected as previously described[41]. Briefly, a nasosorption device ((Hunt Developments UK Ltd) was inserted into the nasal cavity, and the nostril was manually held closed for 60 seconds. The swab was placed in 300 µl of AB-33K (PBS containing 1% BSA and 0.4% Tween-20) and vortexed for 30 seconds. The swab and liquid were placed on a spin filter (Agilent, 5185-5990) and spun at 16,000 rpm for 20 min. Filtered liquid was aliquoted and stored at -80°C. Nasal swabs were collected on 0, 1,

bioRxiv preprint doi: https://doi.org/10.1101/2021.01.09.426058; this version posted January 11, 2021. The copyright holder for this preprint (which was not certified by peer review) is the author/funder. This article is a US Government work. It is not subject to copyright under 17 USC 105 and is also made available for use under a CC0 license.

3, 5, and 7 DPI. BAL was performed on 3, 5, and 7 DPI as previously described. For each procedure, 10-30 mL of sterile saline was instilled and the sample was retrieved with manual suction.[42] Necropsy was performed on 7 DPI and the following tissues were collected: cervical lymph node, mediastinal lymph node, nasal mucosa, trachea, all six lung lobes, right and left bronchus, spleen.

Cells and virus

SARS-CoV-2/human/USA/RML-7/2020 (MW127503.1) was obtained from a nasopharyngeal swab obtained on July 19, 2020. Virus propagation was performed in VeroE6 cells in DMEM2. The used virus stock was 100% identical to the initial deposited Genbank sequence and no contaminants were detected. VeroE6 cells were maintained in DMEM supplemented with 10% fetal bovine serum, 1 mM L-glutamine, 50 U/ml penicillin and 50 µg/ml streptomycin (DMEM10). VeroE6 cells were provided by Dr. Ralph Baric. Mycoplasma testing is performed at regular intervals and no mycoplasma was detected.

Virus titration

Tissue sections were weighed and homogenized in 750 µL of DMEM. Virus titrations were performed by end-point titration in VeroE6 cells, which were inoculated with tenfold serial dilutions of virus swab media or tissue homogenates in 96-well plates. Plates were spun down for 1 hour at 1000 rpm. When titrating tissue homogenate, cells were washed with PBS and 100 µl of DMEM2. Cells were incubated at 37°C and 5% CO2. Cytopathic effect was read 6 days later.

Virus neutralization

Sera were heat-inactivated (30 min, 56 °C), after which two-fold serial dilutions were prepared in DMEM2. 100 $TCID_{50}$ of SARS-CoV-2 strain nCoV-WA1-2020 (MN985325.1) was added.

bioRxiv preprint doi: https://doi.org/10.1101/2021.01.09.426058; this version posted January 11, 2021. The copyright holder for this preprint (which was not certified by peer review) is the author/funder. This article is a US Government work. It is not subject to copyright under 17 USC 105 and is also made available for use under a CC0 license.

After 1hr of incubation at 37°C and 5% CO_2, the virus:serum mixture was added to VeroE6 cells. CPE was scored after 6 days at 37°C and 5% CO_2 for 6 days. The virus neutralization titer was expressed as the reciprocal value of the highest dilution of the serum which still inhibited virus replication.

RNA extraction and quantitative reverse-transcription polymerase chain reaction

RNA was extracted from nasal swabs and BAL using the QiaAmp Viral RNA kit (Qiagen) according to the manufacturer's instructions. Tissue was homogenized and extracted using the RNeasy kit (Qiagen) according to the manufacturer's instructions. Viral gRNA[43] and sgRNA[44] specific assays were used for the detection of viral RNA. Five µl RNA was tested with the Rotor-GeneTM probe kit (Qiagen) or Quantstudio (Thermofisher) according to instructions of the manufacturer. Dilutions of SARS-CoV-2 standards with known genome copies were run in parallel.

Expression and purification of SARS-CoV-2 S and receptor binding domain

Protein production was performed as described previously[45,46]. Expression plasmids encoding the codon optimized SARS-CoV-2 full length S and RBD were obtained from Kizzmekia Corbett and Barney Graham (Vaccine Research Center, Bethesda, USA)[47] and Florian Krammer (Icahn School of Medicine at Mt. Sinai, New York, USA)[48]. Expression was performed in Freestyle 293-F cells (Thermofisher), maintained in Freestyle 293 Expression Medium (Gibco) at 37°C and 8% CO_2 shaking at 130 rpm. Cultures totaling 500 mL were transfected with PEI at a density of one million cells per mL. Supernatant was harvested 7 days post transfection, clarified by centrifugation and filtered through a 0.22 µM membrane. The protein was purified using Ni-NTA immobilized metal-affinity chromatography (IMAC) using Ni Sepharose 6 Fast Flow Resin (GE Lifesciences) or NiNTA Agarose (QIAGEN) and gravity flow. After elution the protein was

bioRxiv preprint doi: https://doi.org/10.1101/2021.01.09.426058; this version posted January 11, 2021. The copyright holder for this preprint (which was not certified by peer review) is the author/funder. This article is a US Government work. It is not subject to copyright under 17 USC 105 and is also made available for use under a CC0 license.

buffer exchanged into 10 mM Tris pH8, 150 mM NaCl buffer (S) or PBS (RBD) and stored at -80°C.

ELISA

ELISA was performed as described previously[8]. Briefly, maxisorp plates (Nunc) were coated overnight at 4°C with 100 ng/well S or RBD protein in PBS. Plates were blocked with 100 µl of casein in PBS (Thermo Fisher) for 1hr at RT. Serum serially diluted 2x in casein in PBS was incubated at RT for 1hr. Antibodies were detected using affinity-purified polyclonal antibody peroxidase-labeled goat-anti-monkey IgG (Seracare, 074-11-021) in casein followed by TMB 2-component peroxidase substrate (Seracare, 5120-0047). The reaction was stopped using stop solution (Seracare, 5150-0021) and read at 450 nm. All wells were washed 4x with PBST 0.1% tween in between steps. Threshold for positivity was set at 3x OD value of negative control (serum obtained from non-human primates prior to start of the experiment) or 0.2, whichever one was higher.

Ig subtyping and SARS-CoV-2 specific IgG/IgA quantification

Ig subtyping was performed using the isotyping Panel 1 Human/NHP Kit on the Meso Quickplex (MSD, K15203D). S and RBD antibodies were determined using the V-PLEX SARS-CoV-2 Panel 2 kit (MSD, K15383U and K15385U).

Antibody-dependent complement deposition

11 µl of Red FluoSpheres™ NeutrAvidin™-Labeled Microspheres (ThermoFisher, F8775) were coated with biotinylated S (25 µl at 1 mg/mL) or RBD protein (5 µl at 1 mg/mL) for 2 hours at 37°C, washed twice with PBS, and diluted in 1 mL of PBS. Serum was diluted 10x in RPMI1640 (Gibco). 10 µl of beads, 40 µl RPMI1640 and 50 ul diluted sera was mixed and incubated for 2 hours at 37°C. Guinea pig complement (Cedarlane, CL4051) was diluted 25x in gelatin veronal

bioRxiv preprint doi: https://doi.org/10.1101/2021.01.09.426058; this version posted January 11, 2021. The copyright holder for this preprint (which was not certified by peer review) is the author/funder. This article is a US Government work. It is not subject to copyright under 17 USC 105 and is also made available for use under a CC0 license.

buffer (Boston Bioproducts, IBB-300X), 100 µl was added to the serum: bead complex and incubated at 37°C for 20 min. The serum:bead complex was then washed twice with 15 mM EDTA and incubated with 50 µl FITC-conjugated-anti-C3 antibody (100x in PBS, MP Biomedical, 855385) for 15 min at RT in dark. Serum:bead complexes were washed three times with PBS and analyzed on a BD FACS Symphony A5 (BD Biosciences) flow cytometer using a high throughput sampler within 1 hour of completion of protocol. All samples were run in duplicate. Serum:bead complexes were gated by FSC vs SSC to remove debris, followed by red bead fluorescence gating in the PE channel, and then the geometric mean fluorescent intensity (MFI) in the FITC channel was determined using FlowJo 10 (BD Biosciences) software and analyzed in Graphpad Prism version 8.3.0..

Antibody dependent monocyte cellular phagocytosis

Beads were prepared as described above. Serum was diluted 100x in RPMI1640, 100 µl was mixed with 10 µl beads and incubated at 37°C for 2 hours. Beads were washed once with RPMI1640. THP-1 cells (ATCC, TIB-202) were diluted to 1.25 x 10^5 cells/mL in RPMI1640, 100 µl was added per sample, and incubated at 37°C for 18 hours. Cells were fixed in 10% formalin for 15 min at RT in dark, washed twice with PBS and ran on a BD FACS Symphony A5 (BD Biosciences) flow cytometer using a high throughput sampler. All samples were run in duplicate as described above.

Antibody-dependent NK cell activation

NK-cell activation was assessed using methods similar to those previously described.1,2 Briefly, cells were isolated from 30 mL of heparin-treated whole blood collected from a healthy human donor (NIH IRB 01-I-N055) using the RosetteSepTM Human NK Cell Enrichment Cocktail according to the manufacturer’s instructions (Stem Cell). NK cells were rested overnight at 37°C

bioRxiv preprint doi: https://doi.org/10.1101/2021.01.09.426058; this version posted January 11, 2021. The copyright holder for this preprint (which was not certified by peer review) is the author/funder. This article is a US Government work. It is not subject to copyright under 17 USC 105 and is also made available for use under a CC0 license.

in complete RPMI 1640 media supplemented with 10% fetal bovine serum and 1 ng/mL of IL-15 (Stem Cell). Nunc MaxiSorpTM 96-well ELISA plates (Thermo Fisher) were coated with 3 µg/mL of SARS-CoV-2 S or RBD antigen for 2 hours at 37°C. Plates were subsequently washed and blocked with a solution of 5% BSA in 1X DPBS overnight at 4oC.

Sera samples collected at -56 and 0 DPI were diluted 1:25 in blocking buffer, plated in duplicates, and incubated on the coated ELISA plates for 2 hours at 37°C. NK cells were resuspended in a staining cocktail containing anti-CD107a-PE/Cy7 antibody (BioLegend), GolgiStop (BD), and GolgiPlug (BD). After removal of sera from the plate, 5.0×10^4 NK cells were added per well and incubated at 37°C for 6 hours.

Surface staining was carried out using anti-CD56-BUV737 (BD), anti-CD16-BV510 (BioLegend), and anti-CD3-BV650 (BD) antibodies prior to fixation and permeabilization using Cytofix/CytoPermTM solution (BD). Intracellular staining was performed using anti-IFNɣ-PerCP/Cy5.5 (BioLegend) and anti-MIP-1β-PE (BD) antibodies. Data acquisition was performed using FACSymphonyTM A5 (BD). NK cells were identified by gating on CD3- CD16+ CD56+ cells.

Integrated analysis of multivariate antibody and virology profiles

Principal component analysis was performed using the R packages "FactoMineR" and "factoextra" to compare antibody and virology profiles. Spearman rank (two-sided) correlation coefficients for pairwise comparisons between all variables were generated using the R "cor" function; the correlation matrix was visualized in R using "ggcorplot."

cDNA Synthesis

cDNAs were prepared according to Briese *et al.*[49] Briefly, RNA was extracted from hamster swabs and tissues following the QiaAmp Viral RNA extraction protocol (Qiagen, Germantown,

bioRxiv preprint doi: https://doi.org/10.1101/2021.01.09.426058; this version posted January 11, 2021. The copyright holder for this preprint (which was not certified by peer review) is the author/funder. This article is a US Government work. It is not subject to copyright under 17 USC 105 and is also made available for use under a CC0 license.

MD) and 11 µL was taken into the SuperScript IV First-Strand cDNA synthesis system (ThermoFisher Scientific, Waltham, MA) following the manufacture's recommendations. After RNase H treatment, second-strand synthesis was performed using Klenow fragment (New England Biolabs, Ipswich, MA) following the manufacturer's recommendations. The resulting double-stranded cDNAs (ds-cDNA) were then purified using Ampure XP bead purification (Beckman Coulter, Pasadena, CA) and eluted into 30 µL water.

Sequencing Library Construction and SARS-CoV2 Enrichment

To construct sequencing libraries, 25 µL ds-cDNA was brought to a final volume of 53 µL in Elution Buffer (Agilent Technologies, Santa Clara, CA) and sheared on the Covaris LE220 (Covaris, Woburn, MA) to generate an average size of 180-220 bp. The following settings were used: peak incident power, 450 watts; duty factor, 15%; cycles per burst, 1000; and time, 300 seconds. The Kapa HyperPrep kit was utilized to prepare libraries from 50 µL of each sheared cDNA sample following modifications of the Kapa HyperPrep kit, version 8.20, and SeqCap EZ HyperCap Workflow, version 2.3, user guides (Roche Sequencing Solutions, Inc., Pleasanton, CA). Adapter ligation was performed for 1 hour at 200C using the Kapa Unique-Dual Indexed Adapters diluted to 1.5 µM concentration (Roche Sequencing Solutions, Inc., Pleasanton, CA). Following ligation, samples were purified with AmPure XP beads (Beckman Coulter, Brea, CA) and subjected to double-sided size selection as specified in the SeqCap EZ HyperCap Workflow User's guide. Pre-capture PCR amplification was performed using 12 cycles, followed by purification using AmPure XP beads.

Purified libraries were assessed for quality on the Bioanalyzer 2100 using the High Sensitivity DNA chip assay (Agilent Technologies, Santa Clara, CA). Quantification of pre-capture

bioRxiv preprint doi: https://doi.org/10.1101/2021.01.09.426058; this version posted January 11, 2021. The copyright holder for this preprint (which was not certified by peer review) is the author/funder. This article is a US Government work. It is not subject to copyright under 17 USC 105 and is also made available for use under a CC0 license.

libraries was performed using the Qubit dsDNA HS Assay kit and the Qubit 3.0 fluorometer following the manufacturer's instructions (ThermoFisher Scientific, Waltham, MA).

The myBaits Expert Virus bait library was used to enrich samples for SARS-CoV-2 according to the myBaits Hybridization Capture for Targeted NGS, version 4.01, protocol. Briefly, libraries were sorted according to estimated genome copies and pooled to create a combined mass of 2 µg for each capture reaction. Depending on estimated genome copies, two to six libraries were pooled for each capture reaction. Capture hybridizations were performed 16-19 hours at 650C and subjected to 8-14 PCR cycles after enrichment. SARS-CoV-2-enriched libraries were purified and quantified using the Kapa Library Quant Universal qPCR mix in accordance with the manufacturer's instructions. Libraries were diluted to a final working concentration of 1-2 nM, titrated to 20 pM, and sequenced as 2 X 150 bp reads on the MiSeq sequencing instrument using the MiSeq Micro kit version 2 (Illumina, San Diego, CA).

Next Generation Sequencing data analysis

Raw fastq reads were adapter trimmed using Cutadapt v 1.12[50], followed by quality trimming and quality filtering using the FASTX Toolkit (Hannon Lab, CSHL). Reads were paired up and aligned to the SARS-CoV-2 genome from isolate SARS-CoV-2/human/USA/RML-7/2020 (MW127503.1) using Bowtie2 v 2.2.9[51]. PCR duplicates were removed using Picard MarkDuplicates v 2.18.7 (Broad Institute). Variant detection was performed using GATK HaplotypeCaller v 4.1.2.0[52] with ploidy set to 2. Raw variant calls were filtered for high confidence variants using bcftools filter[53] with parameters QUAL > 500 and DP > 20.

Histology and immunohistochemistry

Necropsies and tissue sampling were performed according to IBC-approved protocols. Lungs were perfused with 10% neutral-buffered formalin and fixed for eight days. Hereafter, tissue was

bioRxiv preprint doi: https://doi.org/10.1101/2021.01.09.426058; this version posted January 11, 2021. The copyright holder for this preprint (which was not certified by peer review) is the author/funder. This article is a US Government work. It is not subject to copyright under 17 USC 105 and is also made available for use under a CC0 license.

embedded in paraffin, processed using a VIP-6 Tissue Tek (Sakura Finetek, USA) tissue processor, and embedded in Ultraffin paraffin polymer (Cancer Diagnostics, Durham, NC). Samples were sectioned at 5 µm, and resulting slides were stained with hematoxylin and eosin. an in-house SARS-CoV-2 nucleocapsid protein rabbit antibody (Genscript) at a 1:1000 dilution was utilized to detect specific anti-CoV immunoreactivity, carried out on a Discovery ULTRA automated staining instrument (Roche Tissue Diagnostics) with a Discovery ChromoMap DAB (Ventana Medical Systems) kit. The tissue slides were examined by a board-certified veterinary anatomic pathologist blinded to study group allocations. 18 sections, taken from six different lung lobes are evaluated for each animal; a representative lesion from each group was selected for the figure.

Statistics

Two-tailed Mann–Whitney tests, two-way ANOVA, mixed-effect analysis, Fisher test, Spearman rank (two-sided) correlation coefficients, or Kruskall-Wallis analysis were conducted to compare differences between groups using Graphpad Prism version 8.3.0. Statistical tests used are identified in figure legends or main text.

bioRxiv preprint doi: https://doi.org/10.1101/2021.01.09.426058; this version posted January 11, 2021. The copyright holder for this preprint (which was not certified by peer review) is the author/funder. This article is a US Government work. It is not subject to copyright under 17 USC 105 and is also made available for use under a CC0 license.

Table S1. Pathology and IHC scoring direct challenge hamsters. H&E was scored as follows: 0 = not present; 1 = 1-10%; 2 = 11-25%; 3 = 26-50%; 4 = 51-75%; 5 = 76-100%. IHC was scored as follows: 0 = not present; 1 = rare/few; 2 = scattered; 3 = moderate; 4 = numerous; 5 = diffuse.

		IN-vaccinated animals				IM-vaccinated animals				Control animals			
H&E	Lesions %	0	0	0	0	0	0	0	0	70	60	40	70
	Interstitial pneumonia	0	0	0	0	0	0	0	0	4	4	3	4
	Bronchiolitis	0	0	0	0	0	0	0	0	2	2	3	3
	Alveolar exudate	0	0	0	0	0	0	0	0	2	2	2	2
	Type II pneumocyte hyperplasia	0	0	0	0	0	0	0	0	2	2	3	2
	Perivascular leukocyte infiltration	0	0	0	0	0	0	0	0	2	2	2	2
	Edema	0	0	0	0	0	0	0	0	2	2	1	3
IHC	Staining %	0	0	0	0	0	0	0	0	70	60	20	70
	Type I and II pneumocytes	0	0	0	0	0	0	0	0	4	4	3	4
	Exudate	0	0	0	0	0	0	0	0	1	0	1	1
	Bronchiolar epithelium	0	0	0	0	0	0	0	0	3	1	2	3

bioRxiv preprint doi: https://doi.org/10.1101/2021.01.09.426058; this version posted January 11, 2021. The copyright holder for this preprint (which was not certified by peer review) is the author/funder. This article is a US Government work. It is not subject to copyright under 17 USC 105 and is also made available for use under a CC0 license.

Table S2. Pathology and IHC scoring transmission hamsters. H&E was scored as follows: 0 = not present; 1 = 1-10%; 2 = 11-25%; 3 = 26-50%; 4 = 51-75%; 5 = 76-100%. IHC was scored as follows: 0 = not present; 1 = rare/few; 2 = scattered; 3 = moderate; 4 = numerous; 5 = diffuse.

		IN-vaccinated animals				IM-vaccinated animals				Control animals			
H&E	Lesions %	0	0	0	0	0	20	10	5	50	50	50	40
	Interstitial pneumonia	0	0	0	0	0	3	3	2	3	3	3	3
	Bronchiolitis	0	0	0	0	0	0	0	0	3	2	2	2
	Alveolar exudate	0	0	0	0	0	2	1	2	2	3	2	2
	Type II pneumocyte hyperplasia	0	0	0	0	0	3	1	0	2	2	1	1
	Perivascular leukocyte infiltration	0	0	0	0	0	0	2	0	1	1	1	2
	Edema	0	0	0	0	0	0	0	0	3	1	2	2
IHC	Staining %	0	0	0	0	0	5	5	5	60	60	50	30
	Type I and II pneumocytes	0	0	0	0	0	1	1	1	4	4	4	2
	Exudate	0	0	0	0	0	2	0	0	1	1	1	1
	Bronchiolar epithelium	0	0	0	0	0	1	1	0	3	1	2	3

bioRxiv preprint doi: https://doi.org/10.1101/2021.01.09.426058; this version posted January 11, 2021. The copyright holder for this preprint (which was not certified by peer review) is the author/funder. This article is a US Government work. It is not subject to copyright under 17 USC 105 and is also made available for use under a CC0 license.

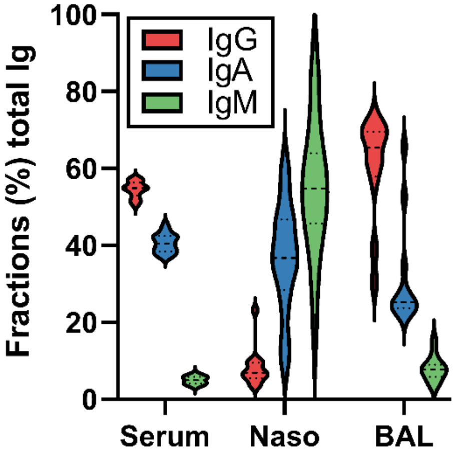

Figure S1. Fractions of IgA, IgG, and IgM in serum, nasosorption or BAL samples.
Twenty-four serum, 14 nasosorption, and 12 BAL samples were investigated.

bioRxiv preprint doi: https://doi.org/10.1101/2021.01.09.426058; this version posted January 11, 2021. The copyright holder for this preprint (which was not certified by peer review) is the author/funder. This article is a US Government work. It is not subject to copyright under 17 USC 105 and is also made available for use under a CC0 license.

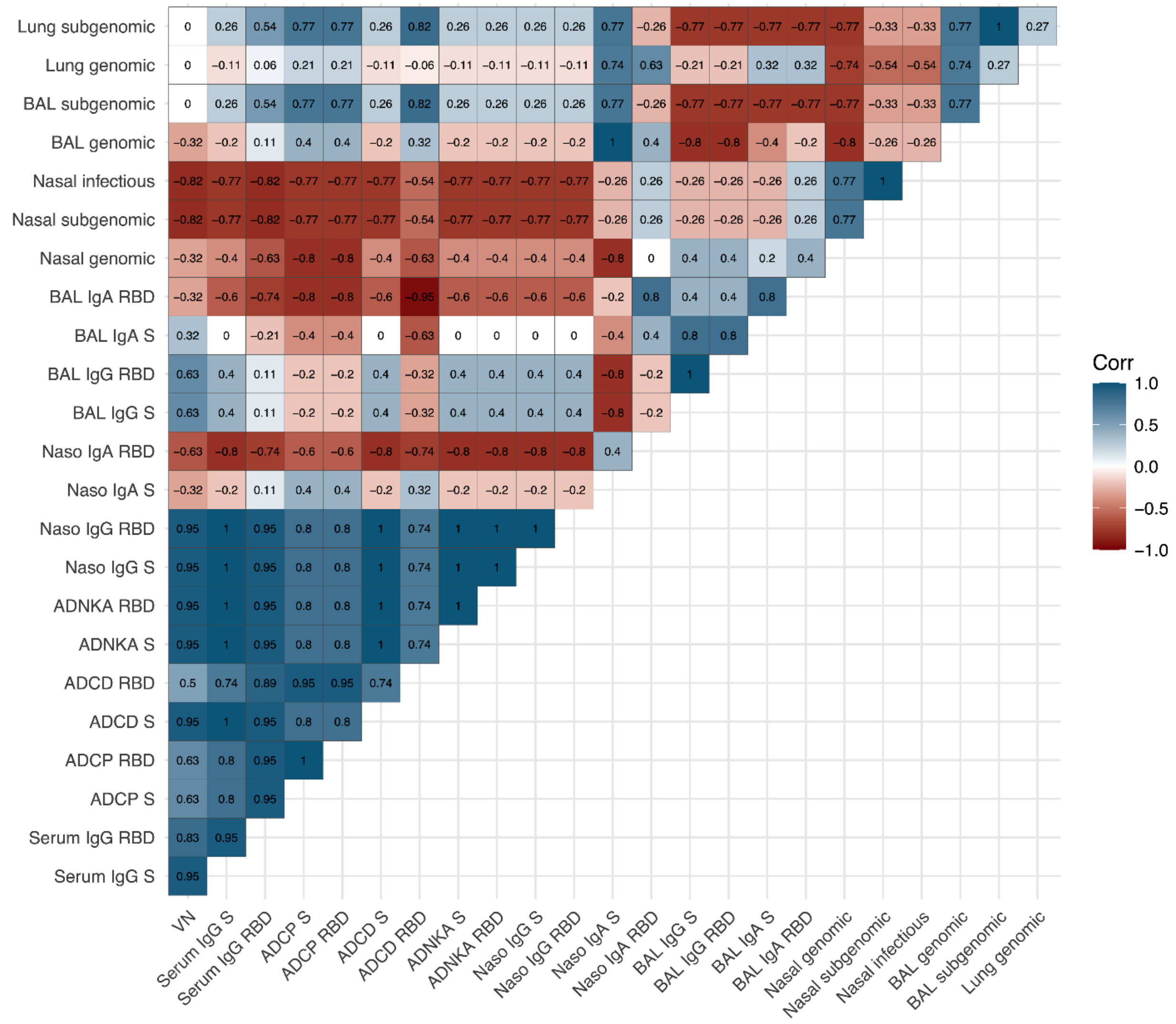

Figure S2. Correlation matrix featuring all immunology and virology measures. Correlation heatmap, depicted as a matrix, representing pairwise correlations between all antibody and virology variables in IN-vaccinated animals. The two-sided Spearman rank correlation coefficient is indicated within each square.
